# Post‐translational modifications of Arabidopsis E3 SUMO ligase AtSIZ1 are controlled by environmental conditions

**DOI:** 10.1002/2211-5463.12309

**Published:** 2017-09-18

**Authors:** Joo Yong Kim, Jong Tae Song, Hak Soo Seo

**Affiliations:** ^1^ Department of Plant Science Research Institute for Agriculture and Life Sciences, and Plant Genomics and Breeding Institute Seoul National University Korea; ^2^ School of Applied Biosciences Kyungpook National University Daegu Korea; ^3^ Bio‐MAX Institute Seoul National University Korea

**Keywords:** abiotic stress, AtSIZ1, COP1, ESD4, sumoylation, ubiquitination

## Abstract

Sumoylation regulates numerous cellular functions in plants as well as in other eukaryotic systems. However, the regulatory mechanisms controlling E3 small ubiquitin‐related modifier (SUMO) ligase are not well understood. Here, post‐translational modification of the Arabidopsis E3 SUMO ligase AtSIZ1 was shown to be specifically controlled by abiotic stresses. AtSIZ1 ubiquitination was induced by exposure to heat stress in transgenic plants overexpressing the E3 ubiquitin ligase COP1. In addition, AtSIZ1 ubiquitination was strongly enhanced in transgenic plants overexpressing SUMO isopeptidase ESD4 under heat stress. By contrast, drought stress induced sumoylation rather than ubiquitination of AtSIZ1 and sumoylated forms of AtSIZ1 accumulated in *esd4* and *cop1–4* mutants. Moreover, *siz1* mutants were found to be tolerant to heat and drought stresses. Taken together, these results indicate that ubiquitination and sumoylation of AtSIZ1 in response to abiotic stresses depend on the activities of COP1 and ESD4 and that the activity and stability of AtSIZ1 can be specifically controlled by different abiotic stresses.

**Abbreviations**
AtSIZ1: Arabidopsis SIZ1
COP1: constitutive photomorphogenic 1
ESD4: early in short days 4
HA: hemagglutinin
PIAL: protein inhibitor of activated stat like
SIZ1: SAP and Miz‐finger domain‐containing protein 1
SUMO: small ubiquitin‐related modifier
XVE: estradiol‐inducible promoter

As sessile organisms, plants are highly susceptible to adverse changes in their environment, and thus modulate their developmental programs to adapt to severe biotic and abiotic stresses. Plants employ numerous mechanisms to adapt to environmental change. One such response mechanism is post‐translational modification, in which molecules such as methyl and phosphate groups, ubiquitin, and small ubiquitin‐related modifier (SUMO) are added to target proteins, impacting their stability and function.

Ubiquitin is a small polypeptide found in almost all tissues in eukaryotic organisms. Ubiquitination, which is the covalent attachment of ubiquitin to a substrate protein, occurs via the sequential activities of ubiquitin‐activating enzyme (E1), ubiquitin‐conjugating enzyme (E2), and ubiquitin ligase (E3) 1. Ubiquitination has a range of effects on target proteins and is involved in regulating cellular localization and activity of target proteins, protein–protein interactions, endocytotic trafficking, inflammation, translation, division and growth, signal transduction, apoptosis, and DNA repair 2, 3, 4, 5, 6, 7. Ubiquitination effects are mediated by two different ubiquitination pathways: monoubiquitination and polyubiquitination. Monoubiquitination, which is the addition of a single ubiquitin molecule to a single residue of the target protein, regulates important cellular functions such as membrane trafficking, endocytosis, viral budding, and histone processes 5, 7, 8, 9. Polyubiquitination is the formation of an ubiquitin chain on a single lysine residue on the target protein. Ubiquitin has seven lysine (K) residues (K6, K11, K27, K29, K33, K48, and K63), all of which can form an isopeptide linkage with an ubiquitin protein 10. K48‐linked chains were the first to be identified and mainly involved in the degradation of proteins via the proteasome 10, which remains the best characterized type of ubiquitin chain. K63 chains are also well understood, but the functions of other lysine chains, mixed chains, branched chains, and heterologous chains (mixtures of ubiquitin and other ubiquitin‐like proteins) remain unclear 9, 10, 11, 12, 13. In most cases, polyubiquitinated proteins are targeted for degradation by the 26S proteasome complex 2, 7, 14. However, monoubiquitination and K63 polyubiquitination are involved in regulating protein activation and signal transduction 9. Ubiquitination is reversed through the action of a large family of deubiquitinating enzymes that control the cellular flux of ubiquitin through its removal from target proteins 15.

Small ubiquitin‐related modifier is another small polypeptide that is covalently attached to target proteins to modify their functions. SUMO, which has ~ 100 amino acids, folds into a similar globular structure as ubiquitin, despite their sharing only 8–15% identity 16. Sumoylation occurs as a result of the activities of three enzymes E1, E2, and E3, which is an enzymatic cascade analogous to ubiquitination 17, 18, 19. By contrast with ubiquitin, SUMO is not usually used to tag proteins for degradation. Instead, sumoylation affects protein subcellular localization, protein function and stability, nuclear–cytosolic transport, transcriptional regulation, apoptosis, stress responses, cell cycle progression, mitochondrial dynamics, and the response to DNA damage 20, 21, 22, 23.

SAF‐A/B‐Acinus‐PIAS (SAP)and Miz‐finger domain‐containing protein 1 (SIZ1) is an E3 SUMO ligase that has a RING‐like domain, Siz‐PIAS RING (SP‐RING), and a chromatin organization domain, SAP 24. Arabidopsis E3 SUMO ligase is involved in several developmental processes including germination, nutrient assimilation, hormone signaling, and flowering 25, 26, 27, 28, 29, 30, 31, 32, 33, 34. In addition, plant E3 SUMO ligases have important roles in the responses to abiotic stresses such as low temperature, drought, heat, and high salt 35, 36, 37, 38, 39, 40.

Interestingly, polysumoylated proteins were also ubiquitinated by SUMO‐targeted ubiquitin ligases (STUbLs) in Arabidopsis, yeast, and humans 41, 42, 43, 44, 45, indicating links between the sumoylation and ubiquitination systems. Moreover, Arabidopsis protein inhibitor of activated stat like1 (PIAL1) and PIAL2 enhanced SUMO chain formation in SUMO‐conjugating enzyme SCE1 46 as E4‐type SUMO ligases 47.

Small ubiquitin‐related modifier is recycled by removal from substrate proteins through the action of SUMO isopeptidases. The Arabidopsis genome contains seven genes encoding SUMO isopeptidases, all of which have specific SUMO isopeptidase activities for different SUMO isoforms 48. The loss of the SUMO isopeptidase early in short days 4 (ESD4) leads to an overaccumulation of SUMO conjugates, early flowering, and severe dwarfism 49. SUMO isopeptidases are involved in flowering, growth, and the response to salt stress 49, 50, 51, 52.

Constitutive photomorphogenic 1 (COP1) is an E3 ubiquitin ligase that consists of a RING‐finger motif, a coiled‐coil domain, and WD40 repeats, and functions as a dimer 53. COP1 polyubiquitinates a range of target proteins, including transcription factors and photoreceptors, inducing their degradation by the 26S proteasome complex and thereby regulating a range of plant growth and development processes such as photomorphogenic responses to far‐red, red, blue, and ultraviolet‐B light, organ development, responses to stress, flowering, and crosstalk between light and hormone signaling 33, 54, 55, 56, 57.

Recent studies reported that Arabidopsis SIZ1 (AtSIZ1) was polyubiquitinated by COP1 and then degraded by the 26S proteasome complex 33, 56. However, the regulatory mechanisms underlying E3 SUMO ligases, including AtSIZ1, are not yet clearly understood.

To further understand E3 SUMO ligase regulation, we examined AtSIZ1 modification under different environmental conditions. AtSIZ1 was ubiquitinated in response to heat stress and sumoylated in response to drought. AtSIZ1 modification was dependent on the activities of COP1 and ESD4. In addition, *siz1–2* and *siz1–3* mutants were tolerant to both heat and drought stresses.

## Materials and methods

### Plant growth conditions and stress treatments

*Arabidopsis thaliana* ecotype Col‐0, *cop1–4*, COP1 transgenic plants, and dominant‐negative (DN)‐COP1 transgenic plants were grown for 10 days on solid MS medium at 22 °C under a 16‐h light/8‐h dark lighting regime. *cop1–4* and COP1 transgenic plants were kindly provided by N.‐H. Chua (Rockefeller University, USA). In preparation for heat treatments, plants were grown as above and then transferred to liquid medium for 2 days to allow adaptation. To initiate heat stress, plants were transferred to liquid culture medium preheated at 37 °C for the indicated time. For drought stress, plants grown on MS were directly exposed to air at 22 °C under continuous light.

### Analysis of the effects of heat stress on the level of AtSIZ1 *in vivo*

Ten‐day‐old plants carrying *XVE‐COP1‐Myc*_*6*_ 58 or *XVE‐DN‐COP1‐Myc*_*6*_ 59 were grown on MS medium in the light for 15 h, with or without 10 μm β‐estradiol (Sigma, St. Louis, MO, USA), and were then transferred to liquid medium preheated at 37 °C for the indicated time. Samples were collected and ground in liquid nitrogen. AtSIZ1, COP1‐Myc_6_ and DN‐COP1‐Myc_6_ levels were examined by western blot analysis using an anti‐AtSIZ1 antibody 33 or an anti‐Myc antibody (Santa Cruz Biotechnology, Dallas, TX, USA).

### Analysis of the effects of ESD4 on the level of AtSIZ1 *in vivo*

The effect of SUMO isopeptidase was examined using total protein extracts from 10‐day‐old wild‐type and *esd4*‐mutant plants grown on MS medium. AtSIZ1 was detected by western blot analysis using an anti‐AtSIZ1 antibody. To further investigate the effect of heat stress on AtSIZ1, transgenic plants overexpressing SUMO isopeptidase ESD4 were produced. A full‐length *ESD4* cDNA was cloned into the plant expression vector pBA002‐HA_4_ and the resulting recombinant plasmid, *35S‐ESD4‐HA*_*4*_, was introduced into Arabidopsis using the floral dip method 60. Ten‐day‐old wild‐type and ESD4‐overexpressing transgenic plants were grown on MS medium and then transferred to liquid media preheated at 37 °C. After treatment for 3 min, samples were collected and ground in liquid nitrogen. AtSIZ1 and ESD4 levels were examined by western blot analysis using an anti‐AtSIZ1 antibody 33 or an anti‐hemagglutinin (HA) antibody (Santa Cruz Biotechnology).

### Analysis of the effects of drought stress on the level of AtSIZ1 *in vivo*

Ten‐day‐old wild‐type plants were grown on MS medium and then exposed to air for 4 h at 22 °C. The plants were collected and total proteins were extracted from the samples. AtSIZ1 level was examined by western blot analysis using an anti‐AtSIZ1 antibody. To examine the effect of SUMO isopeptidase on the level of AtSIZ1 during drought stress, total proteins were extracted from wild‐type and *esd4* mutants grown on MS medium and AtSIZ1 levels were examined by western blot analysis using an anti‐AtSIZ1 antibody 33.

### Determination of the level of AtSIZ1 in *cop1–4* mutants

Ten‐day‐old wild‐type and *cop1–4* plants grown on MS medium were ground in liquid nitrogen, and equivalent amounts were analyzed by western blot analysis using an anti‐AtSIZ1 antibody. To examine the effect of proteasome inhibition on the level and sumoylation of AtSIZ1, 10‐day‐old wild‐type and *cop1–4* mutants grown on MS medium were treated with 50 μm MG132 (Calbiochem, San Diego, CA, USA) for 15 h. Total proteins were extracted from the samples, and AtSIZ1 was detected by western blot analysis using an anti‐AtSIZ1 antibody 33.

### Purification and detection of SUMO1 conjugates

DNA sequences encoding His_6_ (6 × histidine) and 4 × HA were inserted upstream of Arabidopsis *SUMO1* cDNA, and the resulting recombinant *His*_*6*_ *‐HA*_*4*_ *‐SUMO1* DNA was introduced into the β‐estradiol‐inducible vector pER8 61. The construct was transformed into Arabidopsis by the floral dip method 60 to generate a SUMO1‐overexpressing Arabidopsis line. SUMO1 conjugates were assessed in plants carrying the *XVE‐His*_*6*_ *‐HA*_*4*_ *‐SUMO1* transgene. Plants were grown on MS media for 2 weeks before being treated with 10 μm β‐estradiol for 15 h under light conditions and then being directly exposed to air for 4 h. Samples were harvested, ground in liquid nitrogen, and resuspended in extraction buffer (20 mm Tris/HCl pH 8.0, 8 m urea, 100 mm NaH_2_PO_4_, 1% Triton X‐100, 10 mm β‐mercaptoethanol) containing 1× protease inhibitor cocktail without EDTA (Roche, Basel, Basel‐Stad, Switzerland) and 20 mm imidazole (Sigma). After centrifugation, supernatants were purified on Ni^2+^‐NTA columns using a 20–500 mm imidazole concentration gradient, according to the manufacturer's instructions (Qiagen, Hilden, North Rhine‐Westphalia, Germany). Eluted proteins were detected by western blot analysis with an anti‐HA antibody (Santa Cruz Biotechnology) or an anti‐AtSIZ1 antibody 33.

### Analysis of the effect of heat on the growth of *siz1* mutant

To assess heat tolerance, 5‐day‐old or 2‐week‐old wild‐type, *siz1*–*2*, and *siz1*–*3* seedlings germinated and grown on MS medium were used. To test 5‐day‐old plants, seeds of wild‐type, *siz1*–*2,* and *siz1*–*3* mutants were sown on MS media and grown for 5 days at 22 °C, and then, the plants were treated in a water bath in three different ways. First, the plants were heated at 45 °C for 1 h. After treatment, the plants were further incubated at 22 °C for 5 days and then photographed. Second, the plants were heated at 45 °C for 90 min. After treatment, the plants were further incubated at 22 °C for 5 days and then photographed. Third, the plants were heated at 37 °C for 1 h and then incubated at 22 °C for 1 h. After further treatment at 45 °C for 180 min, the plants were incubated at 22 °C for 5 days and then photographed. To test 2‐week‐old plants, seeds of wild‐type, *siz1*–*2,* and *siz1*–*3* mutants were sown on MS media and grown for 2 weeks at 22 °C, and then, the plants were treated in a water bath in three different ways. First, the plants were heated at 45 °C for 40 min. After treatment, the plants were further incubated at 22 °C for 1 week and then photographed. Second, the plants were heated at 37 °C for 1 h and then incubated at 22 °C for 1 h. After further treatment at 45 °C for 40 min, the plants were incubated at 22 °C for 1 week and then photographed. Third, the plants were heated at 37 °C for 1 h and then incubated at 22 °C for 1 h. After further treatment at 45 °C for 160 min, the plants were incubated at 22 °C for 1 week and then photographed. A consistent number of plants were grown per tray to minimize experimental variations. The heat tolerance of 10‐day‐old wild‐type, *siz1*–*2*, and *siz1*–*3* seedlings was also examined according to the method which was previously published 35.

### Analysis of the effect of drought on the growth of *siz1* mutants

To assess drought tolerance in soil‐grown plants, seeds of wild‐type, *siz1*–*2,* and *siz1*–*3* mutants were directly sown in the soil, and plants were then subjected to progressive drought by withholding water for 30 days. The plants were then watered for 5 days and photographed. A consistent number of plants were grown per tray to minimize experimental variations.

## Results

### AtSIZ1 can be modified by COP1 activity under heat stress

**Figure 1:**
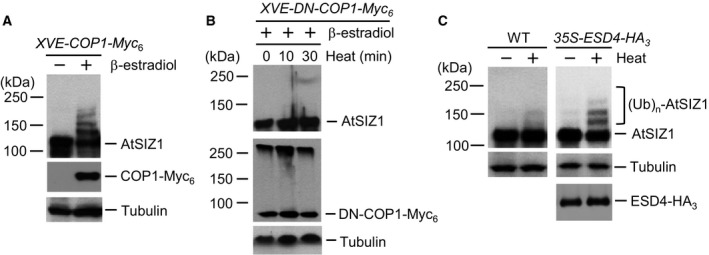
Heat‐induced AtSIZ1 modification is regulated by COP1 and ESD4 activity. (A) Transgenic *XVE‐COP1‐Myc*_*6*_ plants were incubated in liquid medium with β‐estradiol to induce COP1 expression. After incubation for 15 h, the plants were treated with liquid MS medium preheated to 37 °C. Samples were collected after treatment for 30 min, and AtSIZ1 and COP1‐Myc_6_ were detected by western blot analysis with anti‐AtSIZ1 or anti‐Myc antibodies. Tubulin was used as a loading control. (B) Transgenic *XVE‐DN‐COP1‐Myc*_*6*_ plants were incubated in liquid medium with β‐estradiol to induce DN‐COP1 expression. After incubation for 15 h, the plants were treated with liquid MS medium preheated to 37 °C. Samples were collected after treatment for 0, 10, and 30 min, and AtSIZ1 and DN‐COP1‐Myc_6_ were detected by western blot analysis with anti‐AtSIZ1 or anti‐Myc antibodies. Tubulin was used as a loading control. (C) Ten‐day‐old wild‐type and ESD4‐overexpressing transgenic plants grown in MS medium were treated with liquid MS medium preheated at 37 °C. After treatment for 30 min, total proteins were extracted and AtSIZ1 was detected by western blot analysis with anti‐AtSIZ1 or anti‐HA antibodies. Tubulin was used as a loading control.

Previous research showed that numerous proteins were modified by SUMO under conditions of heat stress 35, 62, 63, 64, 65, 66, 67. However, it is unclear whether E3 SUMO ligase is itself regulated by sumoylation or other protein modifications in response to stresses such as heat. COP1 exhibited E3 ubiquitin ligase activity for AtSIZ1 33, 56, and we used this system to investigate modification of E3 SUMO ligase in response to stress. First, the effect of COP1 on heat‐induced AtSIZ1 ubiquitination was investigated using *XVE*‐*COP1‐Myc*_*6*_ transgenic plants in which COP1 expression was inducible with β‐estradiol. AtSIZ1 levels were reduced by induction via COP1 (Fig. 1A). Instead, modified AtSIZ1 bands appeared upon induction by COP1, but they were not detected in noninduced plants (Fig. 1A). This result was confirmed by examining heat‐induced AtSIZ1 modifications in *XVE*‐*DN*‐*COP1‐Myc*_*6*_ transgenic plants that overexpressed DN‐COP1 upon β‐estradiol induction. However, the modified AtSIZ1 bands were not detected upon induction by DN‐COP1 (Fig. 1B). Previously, we proved that AtSIZ1 was directly modified by ubiquitin through E3 ubiquitin ligase activity of COP1 *in vitro* 33. Current result showed that the pattern of heat stress‐induced AtSIZ1 modification (Fig. 1A) was very similar to the ubiquitination pattern of AtSIZ1 33, suggesting that heat stress‐induced AtSIZ1 modification occurred through E3 ubiquitin ligase activity of COP1 and the modified upper bands may be ubiquitinated AtSIZ1.

### COP1‐dependent AtSIZ1 modification can be enhanced by ESD4 activity under heat stress

Substantial accumulation of SUMO conjugates was previously observed in *esd4* mutants 49. *ESD4* encodes a SUMO isopeptidase, which removes SUMO from SUMO conjugates 49. Therefore, the effect of ESD4 on heat‐induced AtSIZ1 level was also examined using ESD4‐overexpressing transgenic plants that harbored *35S‐ESD4‐HA*_*3*_. Overexpression of ESD4 resulted in a decrease in AtSIZ1 level under heat stress condition (Fig. 1C). Modified AtSIZ1 bands were also clearly appeared upon on overexpression of ESD4 under heat stress condition (Fig. 1C) as shown in induction of COP1 (Fig. 1A). This suggests that ESD4 acts as a SUMO isopeptidase for AtSIZ1 and that desumoylation of AtSIZ1 by ESD4 leads to AtSIZ1 modification by COP1 activity.

### *siz1* mutants are tolerant to heat stress

**Figure 2:**
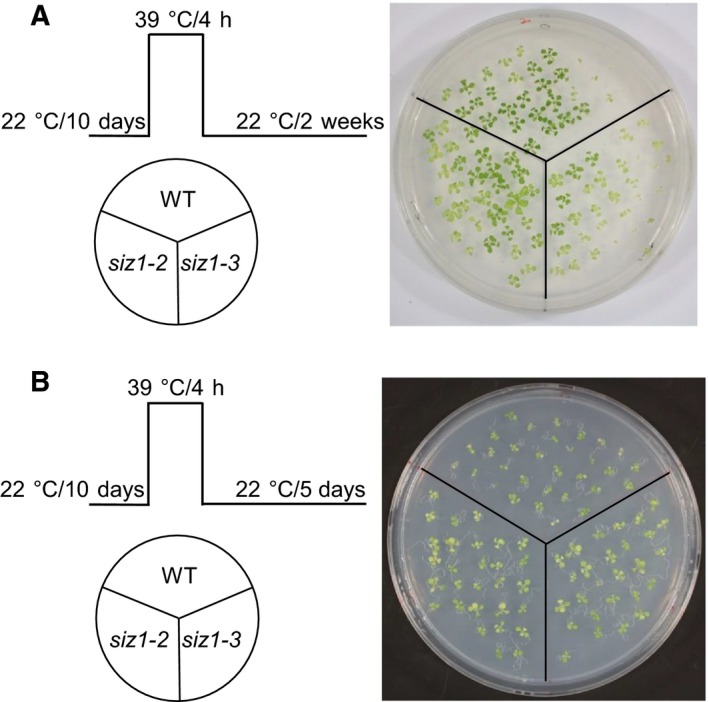
Examination of the heat tolerance of *siz1* mutants. Ten‐day‐old wild‐type, *siz1*–*2*, and *siz1*–*3* seedlings germinated and grown on MS medium were subjected to a heat shock treatment. The phenotypes of the wild‐type, *siz1*–*2*, and *siz1*–*3* seedlings were examined after treatment by different heat shock regimes schematically shown on the left of each section. (A) Heat shock was treated in a water bath. The plants were photographed 2 weeks after heat treatment. (B) Heat shock was treated in a growth chamber. The plants were photographed 5 days after heat treatment.

Numerous proteins are conjugated to SUMO in response to heat stress, suggesting that sumoylation contributes to heat tolerance 35, 62, 63, 64, 65, 66, 67. Here, AtSIZ1 was polyubiquitinated rather than sumoylated under heat stress conditions (Fig. 1). Therefore, we examined whether the loss of AtSIZ1 resulted in positive or negative effects on heat‐stressed plants. First, we tested the heat tolerance of *siz1* mutants according to the method which was previously performed 35. Ten‐day‐old wild‐type, *siz1*–*2*, and *siz1*–*3* mutants were subjected to heat shock treatment. Heat treatment at 39 °C for 4 h was carried out in a water bath or growth chamber. Results showed that there was no difference in heat tolerance between wild‐type and *siz1*–*2* seedlings or between wild‐type and *siz1*–*3* seedlings (Fig. 2A,B). But, these results differ from the previous result that *siz1* mutants are more sensitive to heat stress than wild‐type 35.

**Figure 3:**
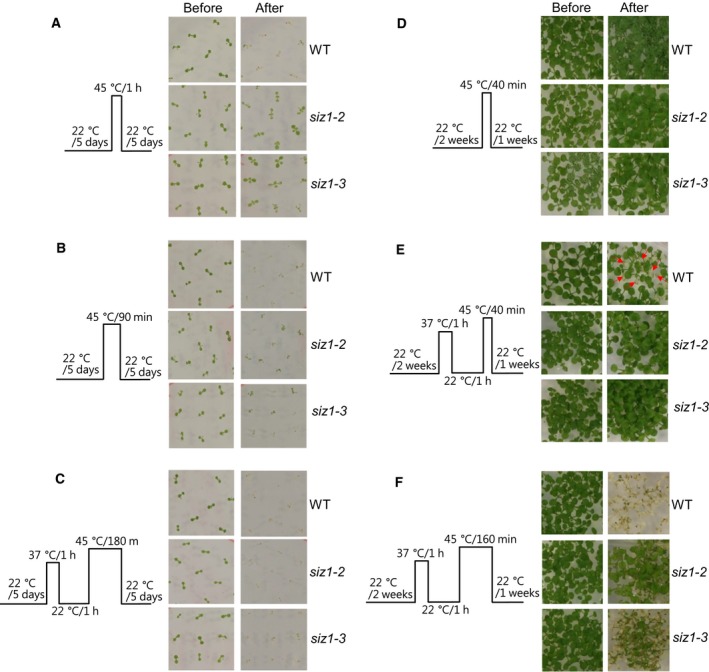
*siz1* mutants are tolerant to heat stress. Five‐day‐old (A–C) or 2‐week‐old (D–F) wild‐type, *siz1*–*2*, and *siz1*–*3* seedlings germinated and grown on MS medium were subjected to a heat shock treatment. The phenotypes of the wild‐type, *siz1*–*2*, and *siz1*–*3* seedlings were examined after treatment by different heat shock regimes schematically shown on the left of each section. The plants were photographed 5 days or 1 week after heat treatment. Arrows indicate chlorotic rosette leaves.

Thus, heat treating method was changed. Five‐day‐old or 2‐week‐old wild‐type, *siz1*–*2*, and *siz1*–*3* mutants were subjected to heat shock treatment in three different ways. In the case of 5‐day‐old seedlings, most of the wild‐type plants developed severe chlorotic symptoms and eventually died, whereas *siz1*–*2* and *siz1*–*3* mutants were still healthy after heat treatment at 45 °C for 1 h (Fig. 3A). But, many of *siz1*–*2* and *siz1*–*3* mutants also developed chlorotic symptoms after heat treatment at 45 °C for 90 min although survival rates of *siz1*–*2* and *siz1*–*3* mutants were higher than those of wild‐type (Fig. 3B). In addition, heat treatment at 45 °C for 180 min after pretreatment at 37 °C for 1 h resulted in death of both the wild‐type and *siz1* mutants (Fig. 3C). In the case of 2‐week‐old seedlings, heat treatment at 45 °C for 40 min had no effect on the growth of wild‐type, *siz1*–*2* and *siz1*–*3* mutants (Fig. 3D). Heat treatment at 45 °C for 40 min after pretreatment at 37 for 1 h resulted in partial rosette leaf chlorosis in wild‐type but not in *siz1*–*2* and *siz1*–*3* mutants (Fig. 3E). More severe condition, heat treatment at 45 °C for 160 min after pretreatment at 37 for 1 h, caused the death of wild‐type, while *siz1*–*2* and *siz1*–*3* mutants were still alive (Fig. 3F). Interestingly, *siz1*–*2* mutants were more resistant than *siz1*–*3* mutants (Fig. 3F).

### Drought stress‐induced AtSIZ1 modification depends on COP1 and ESD4 activity

**Figure 4:**
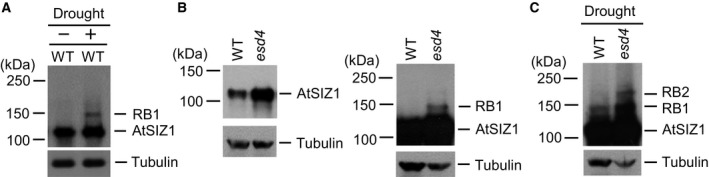
ESD4 induces AtSIZ1 modification during drought stress. (A) Ten‐day‐old wild‐type plants grown in MS medium were dried for 4 h. The plants were collected and total proteins were extracted. AtSIZ1 was detected by western blot analysis with an anti‐AtSIZ1 antibody. Tubulin was used as a loading control. (B) Total proteins were extracted from 10‐day‐old wild‐type and *esd4* plants grown in MS medium, and AtSIZ1 was detected by western blot analysis with an anti‐AtSIZ1 antibody. Blot was exposed to X‐ray film for short (left panel) or long period of time (right panel). (C) Ten‐day‐old wild‐type and *esd4* plants grown in MS medium were dried for 4 h. The plants were collected and total proteins were extracted. AtSIZ1 was detected by western blot analysis with an anti‐AtSIZ1 antibody. Tubulin was used as a loading control.

Previous research showed that several proteins were modified by SUMO under drought stress conditions 33, 36, 65. To assess the effect of drought on AtSIZ1, wild‐type plants were dried in the air for 4 h and AtSIZ1 abundance in tissue samples was assessed by western blot analysis. AtSIZ1 levels were similar in control and drought‐stressed plants (Fig. 4A). An additional protein (upper band), which was named retarded band 1 (RB1), was detected only in drought‐stressed plants (Fig. 4A).

The effect of drought on AtSIZ1 abundance was also examined in *esd4* mutants. AtSIZ1 levels were elevated in *esd4* mutants compared to the wild‐type and only a single protein single band was observed in the original analysis (Fig. 4B, left panel). However, longer exposure of the western blot revealed an additional AtSIZ1 band (Fig. 4B, right panel). Western analysis of AtSIZ1 in air‐dried *esd4* mutants revealed a second additional protein band, named retarded band 2 (RB2), that was larger than RB1 (Fig. 4C).

### AtSIZ1 may be accumulated as sumoylated forms in *esd4* and *cop1*–*4* mutants, respectively

**Figure 5:**
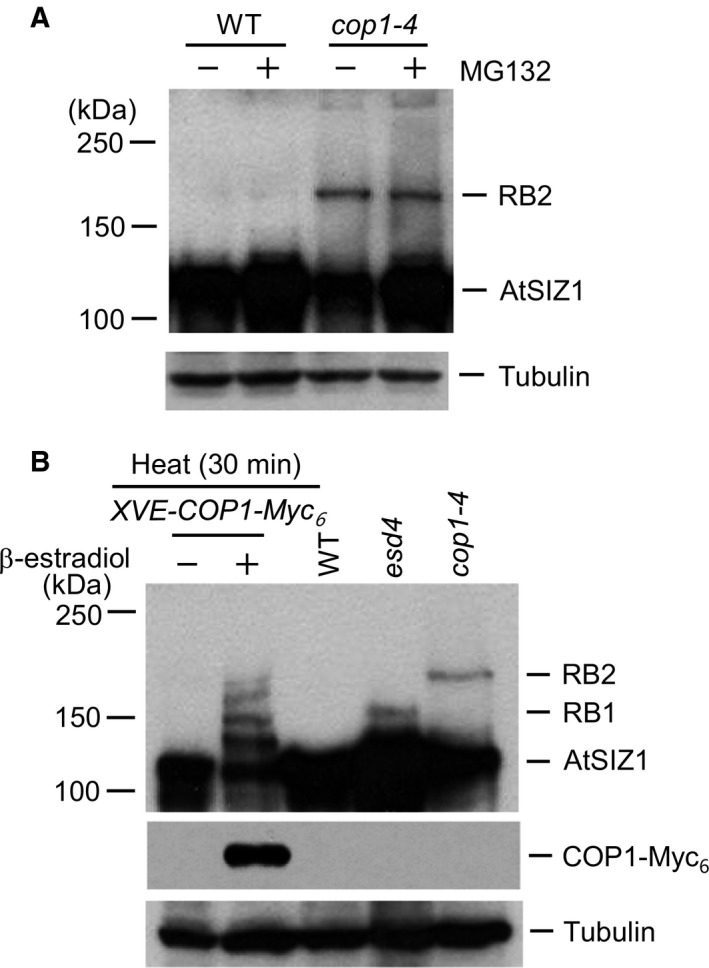
AtSIZ1 can be accumulated as sumoylated forms in *esd4* and *cop1*–*4* mutants. (A) Ten‐day‐old wild‐type and *cop1*–*4* plants were treated with or without MG132. Total proteins were extracted from the samples and AtSIZ1 was detected by western blot analysis with an anti‐AtSIZ1 antibody. Tubulin was used as a loading control. (B) Ten‐day‐old transgenic seedlings harboring *XVE‐COP1‐Myc*_*6*_ were treated with β‐estradiol to induce COP1 expression. After incubation for 15 h, the plants were treated with liquid MS medium preheated to 37 °C. Samples were collected after treatment for 30 min. Total proteins were extracted from wild‐type, *esd4*, and *cop1*–*4* plants and from heat‐treated transgenic plants. AtSIZ1 and COP1‐Myc_6_ were detected by western blot analysis with anti‐AtSIZ1 or anti‐Myc antibodies. Tubulin was used as a loading control.

Our previous study showed that AtSIZ1 was more abundant in *cop1*–*4* mutants than in wild‐type plants under both light and dark conditions 33. As above, the original western blot was exposed to X‐ray film for a relatively short time and only a single AtSIZ1 band was seen. Western blot analysis of AtSIZ1 in *cop1*–*4* mutants was repeated, and an additional upper protein band was observed (Fig. 5A). Upper band intensity was unaffected by treatment with the proteasome inhibitor MG132 (Fig. 5A). Western blot analysis was also performed using *esd4* and *cop1*–*4* mutants, and heat‐treated *XVE‐COP1‐Myc*_*6*_ transgenic plants after treatment of β‐estradiol. RB1 and RB2 were detected in *esd4* and *cop1*–*4* mutants, respectively (Fig. 5B), and the RB1 and RB2 sizes resembled the third and fifth AtSIZ1 bands, respectively, when compared to the AtSIZ1 bands detected in COP1‐Myc_6_‐overexpressing plants (Fig. 5B). Previous studies noted that the migration of ubiquitinated and sumoylated proteins was retarded by ~ 8 68 and 15–20 kDa 69, respectively, during SDS/PAGE analysis, suggesting that RB1 and RB2 may be sumoylated AtSIZ1 bands.

**Figure 6:**
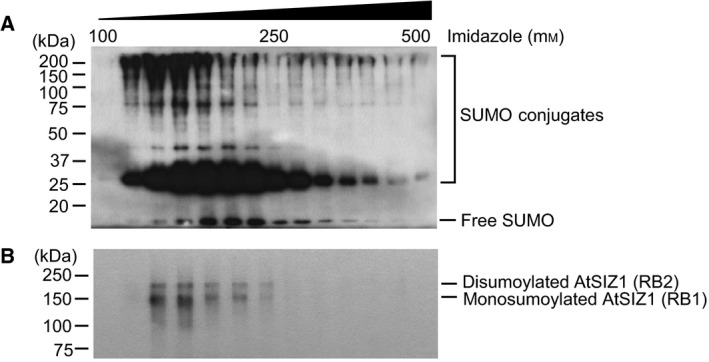
AtSIZ1 is monosumoylated or disumoylated *in vivo*. (A) SUMO conjugates were purified from 10‐day‐old plants carrying *XVE‐His*_*6*_ *‐HA*_*4*_ *‐SUMO1* and detected by western blot analysis with an anti‐HA antibody. (B) The same eluted fractions were examined by western blot analysis with an anti‐AtSIZ1 antibody.

Finally, to confirm whether RB1 and RB2 were sumoylated AtSIZ1, SUMO conjugates were purified and examined for the presence of AtSIZ1. A transgenic plant line (*XVE‐His*_*6*_ *‐HA*_*4*_ *‐SUMO1*) was generated that expressed β‐estradiol‐inducible SUMO1 tagged with N‐terminal His_6_ and HA_4_ for the purification and detection of SUMO1 conjugates by nickel affinity column and western blot analysis, respectively. Transgenic plants expressing His_6_‐HA_4_‐SUMO1 were exposed to drought. Analysis of purified SUMO1 conjugates by western blot using an anti‐HA antibody showed that purification of SUMO1 conjugates was successful (Fig. 6A). Analysis of purified SUMO1 conjugates with an anti‐AtSIZ1 antibody revealed two clear bands (Fig. 6B) that corresponded in size to those detected in *esd4* and *cop1*–*4* mutants (Fig. 5B), strongly indicating that RB1 and RB2 can be sumoylated AtSIZ1.

### *siz1* mutants are tolerant to drought stress

**Figure 7:**
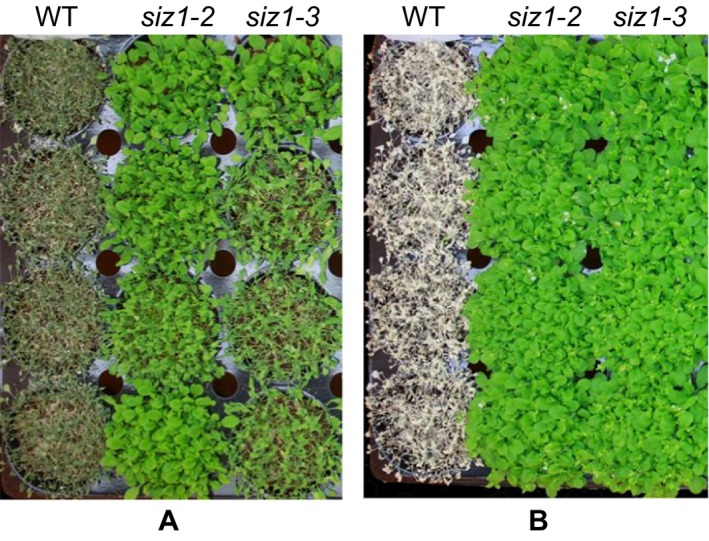
*siz1* mutants are tolerant to drought stress. (A) Wild‐type, *siz1*–*2*, and *siz1*–*3* seeds were sown in soil and watering was withheld for 30 days. (B) Plants were watered for 5 days and then photographed.

Small ubiquitin‐related modifier conjugation is known to stabilize or activate proteins. The induction of AtSIZ1 sumoylation by drought suggested that AtSIZ1 was stabilized or activated under drought conditions. To investigate this, *siz1*–*2* and *siz1*–3 mutants were used to determine whether *siz1* mutants were sensitive or resistant to drought stress. Wild‐type, *siz1*–*2*, and *siz1*–*3* seeds were directly sown in soil and were challenged with drought by withholding water for 30 days (Fig. 7A). The plants were then rewatered and photographed after 5 days (Fig. 7B). Wild‐type plants died, but growth recovered in the *siz1*–*2* and *siz1*–*3* mutants (Fig. 7A and 7B). This suggests that loss of AtSIZ1 confers drought tolerance.

## Discussion

Plant E3 SUMO ligases are involved in responding to abiotic stresses, such as cold, drought, heat, and salt 35, 36, 37, 38, 39, and also have roles in various development processes 25, 26, 27, 28, 29, 30, 31, 32, 34, 56. Recent studies showed that the activity and stability of Arabidopsis E3 SUMO ligase AtSIZ1 were regulated by COP1 activity 33, 56. In this study, the post‐translational regulatory mechanisms controlling AtSIZ1 abundance under conditions of heat and drought stress were examined.

Cold, heat, and drought stresses stimulate the conjugation of SUMO to a range of target proteins 33, 62, 63, 64, 65, 66, 67. This suggested that the stability and activity of E3 SUMO ligase might also be post‐translationally modulated by stress. To test this, post‐translational modification of AtSIZ1 was examined in COP1‐ and ESD4‐overexpressing transgenic lines after exposure to heat or drought stress. AtSIZ1 ladder bands were detected in *XVE*‐*COP1‐Myc*_*6*_ transgenic plants that overexpressed COP1 upon β‐estradiol induction (Fig. 1A). It is well known that ubiquitin conjugates display an increase of ~ 8 kDa after ubiquitination 68, indicating that the ladder bands must be ubiquitinated AtSIZ1 (Fig. 1A). It also indicates that AtSIZ1 must be polyubiquitinated in response to heat stress and ubiquitination is dependent on COP1 activity. Besides, the ladder bands were clearly detected in *35S*‐*ESD4‐HA*_*3*_ transgenic plants exposed to heat (Fig. 1C), strongly suggesting that desumoylation of AtSIZ1 by ESD4 stimulates AtSIZ1 ubiquitination by COP1 under heat stress.

Small ubiquitin‐related modifier conjugation was lower in *siz1* mutants than in wild‐type Arabidopsis plants, even under heat stress 35. Conversely, heat‐induced sumoylation of target proteins was observed in several previous studies 62, 63, 64, 65, 66, 67. In addition, rice E3 SUMO ligases OsSIZ1 and OsSIZ2 were involved in sumoylation of target proteins under heat stress 62, and transgenic Arabidopsis overexpressing tomato E3 SUMO ligase SISIZ1 also accumulated SUMO conjugates in response to heat stress 70. There are three types of E3 SUMO ligase in yeast and animal systems: SIZ/PIAS, RanBP2, and polycomb 71. However, only the SIZ/PIAS type has been identified in plants to date. Homologs of the polycomb and RanBP2 types have not been identified due to a lack of conserved motifs or domains. Our observation that AtSIZ1 ubiquitination is induced by heat suggests that AtSIZ1 is degraded in response to heat stress. It therefore remains unclear how AtSIZ1 participates in the sumoylation of target proteins under heat stress conditions, or why SUMO conjugate levels are lower in *siz1* mutants than in wild‐type plants under heat stress. We propose that 1) AtSIZ1 levels are fine‐tuned in response to heat stress and thereby the degradation of AtSIZ1 is a regulatory mechanism to prevent constitutive sumoylation of certain target proteins during heat stress, or 2) high PLOIDY 2 (HPY2) or other types of E3 SUMO ligase (including polycomb proteins) can also act as E3 SUMO ligases to sumoylate target proteins under heat stress.

Previously, it was reported that *siz1*–*2* and *siz1*–*3* seedlings exhibited thermosensitivity and AtSIZ1 positively controlled the heat stress response 35. However, in the present study, *siz1*–*2* and *siz1*–*3* mutants were not sensitive to heat stress or were resistant to heat stress compared with wild‐type plants (Fig. 2 and Fig. 3). Currently, the reasons for varied responses to heat stress are unclear. Further experiments under various heat treatment conditions and growth stages may be required to explain this inconsistency. Nevertheless, we carefully suggest that AtSIZ1 plays a negative role in the response to heat stress through its E3 SUMO ligase activity.

It appears that no ubiquitination of AtSIZ1 was observed under drought stress (Figs. 4 and 5), although it is still possible that AtSIZ1 ubiquitination occurs under drought stress. Western analysis of drought‐stressed plants revealed two additional protein fragments, termed RB1 and RB2, that were larger in size than AtSIZ1 and which were also detected in *esd4* and *cop1*–*4* mutants (Figs. 4 and 5). Further analysis using a transgenic line overexpressing His_6_‐HA_4_‐SUMO1 strongly indicated that RB1 and RB2 must be monosumoylated and disumoylated AtSIZ1, respectively (Fig. 6B). These data indicate that AtSIZ1 sumoylation can be stimulated by the loss of ESD4 and COP1 and that AtSIZ1 can be activated by drought stress. AtSIZ1 can be accumulated as monosumoylated or disumoylated forms in plant tissues and sumoylation can protect AtSIZ1 from degradation by the 26S proteasome complex after polyubiquitination. There is accumulating evidence that sumoylation is a dynamic process that influences the conformation of the target protein, thereby changing the interaction of the modified protein with other proteins, as well as the subcellular localization, stability, and activity of the modified protein 72, 73, 74. In addition, we previously showed that mutant FLC (K154R, a mutation of the sumoylation site) does not possess flowering repression activity 29 and that transgenic *sly1*–*13* mutants overexpressing SLY1 were phenotypically similar to wild‐type plants, whereas *sly1*–*13* plants overexpressing a mutated mSLY1 protein (K122R, a mutation at the sumoylation site) retained the mutant dwarfing phenotype 31. In addition, the activities of Arabidopsis nitrate reductases NIA1 and NIA2 increased significantly after sumoylation 28. These findings indicate that sumoylation directly activates FLC, SLY1, NIA1, and NIA2 functions. Sumoylation has also been implicated in the control of proteasomal degradation of several substrate proteins 75. For example, sumoylation can oppose ubiquitination and proteasomal degradation by competitively attaching to the same lysine residues within substrate proteins, as is the case for inhibitors of NF‐kappa B (IkB) 76, mouse double minute homolog 2 (MDM2) 77, and serine hydroxyl methyltransferase SHMT1 78. In addition, cyclin‐dependent kinase 6 (CDK6) sumoylation at lysine 216 blocks its ubiquitination at lysine 147 and inhibits ubiquitin‐mediated CDK6 degradation 79. These findings indicate that sumoylation protects IkB, MDM2, SHMT1, and CDK6 from degradation by the 26S proteasome complex after polyubiquitination. Therefore, the current data strongly suggest that sumoylation activates and stabilizes AtSIZ1 under drought stress. Previous studies also observed sumoylation of several proteins in response to drought 33, 34, 67. Our data suggest that AtSIZ1 participates in the sumoylation of target proteins in response to drought stress, working independently or together with other E3 SUMO ligases.

Sumoylation of AtSIZ1 under drought conditions suggested that AtSIZ1 may be involved in the response to drought and prompted the investigation of the effects of dehydration on *siz1* mutant growth. However, *siz1*–*2* and *siz1*–*3* mutants were more drought resistant than wild‐type plants (Fig. 7), indicating that AtSIZ1 negatively controlled the drought stress response. This contrasts with a previous study suggesting that AtSIZ1 was a positive regulator of drought stress tolerance 36, but was consistent with research in which siz1–2 and siz1–3 mutants exhibited drought resistance 80. The reasons for this discrepancy are unclear but may also be related to different growth conditions. Mutation of another E3 SUMO ligase, HPY2, also increased Arabidopsis drought tolerance 81. More research is needed to fully understand the role of E3 SUMO ligase in the response to drought. However, observations of plant responses to heat and drought indicate that E3 SUMO ligases AtSIZ1 and HPY2 have crucial functions in plant adaptations to abiotic stresses.

Our data indicate that modification of Arabidopsis E3 SUMO ligase AtSIZ1 with ubiquitin or SUMO is controlled by the activities of E3 ubiquitin ligase COP1 or SUMO isopeptidase ESD4 during heat and drought stresses. In addition, our study strongly suggests that the activity and stability of AtSIZ1 can be post‐translationally fine‐tuned by various modifiers under conditions of stress. Identification of the specific modifiers that are conjugated to AtSIZ1 under different conditions is needed to fully characterize the regulatory mechanisms underlying control of AtSIZ1 during exposure to biotic and abiotic stress. Investigation of additional post‐translational modifications of E3 SUMO ligases AtSIZ1 and HPY2 will further enhance our understanding of plant growth and development by sumoylation.

## Author contributions

HSS designed the project. JYK and HSS carried out experiments. JYK, JTS, and HSS analyzed and interpreted the data. JYK and HSS wrote the manuscript. All authors commented on the results and the manuscript.
